# Incidental finding of Langerhans cell histiocytosis of temporoparietal bone - A case report

**DOI:** 10.1016/j.ijscr.2021.106179

**Published:** 2021-07-07

**Authors:** Aakash Mishra, Sandesh Gyawali, Sanjeev Kharel, Aman Mishra, Sandip Kuikel, Nibesh Pathak, Ashim Gurung

**Affiliations:** a Kathmandu Medical College Teaching Hospital, Kathmandu, Nepal; b Department of General Surgery, National Academy of Medical Sciences, Bir Hospital, Kathmandu, Nepal; c Maharajgunj Medical Campus, Institute of Medicine, Maharajgunj, Kathmandu, Nepal; d Department of Neurosurgery, Maharajgunj Medical Campus, Institute of Medicine, Kathmandu, Nepal

**Keywords:** Langerhans cell histiocytosis, Temporal bone, Parietal bone, Pediatric

## Abstract

**Introduction and importance:**

Langerhans cell histiocytosis (LCH) is a rare haematological disorder affecting infants and young children and has an estimated incidence of 2-5 cases per million people per year. LCH invades the reticuloendothelial system and causes the proliferation of Langerhans cells and mature eosinophils. LCH involving the temporoparietal bone has rarely been reported in the literature.

**Presentation of case:**

A ten-year-old boy presented to the Neurosurgical outpatient clinic with a swelling on the right temporoparietal region following a fall from his bicycle. Local examination revealed a single, 3 × 3 cm, non-tender, cystic, immobile swelling in the right temporoparietal region. On evaluation for recent head trauma, an incidental finding of eosinophilic granuloma was discovered on a CT scan. The FNAC was suggestive of a histiocytic lesion pertaining to a diagnosis of LCH. The patient underwent wide excision of the mass and cranioplasty. A one-month follow-up CT scan of the head had no evidence of residual or recurrent disease.

**Discussion:**

Eosinophilic granuloma is one of the three variants of LCH and has a relatively better prognosis. Clinical diagnosis can be challenging and mandates tissue sampling for histopathological examination. Treatment modalities including surgery, radiotherapy, chemotherapy, and steroid injection are used alone, or in combination, depending on the extent and severity of the disease.

**Conclusion:**

Examining a swelling in the temporoparietal region with no other characteristic symptoms could be a case of LCH. The timely diagnosis and surgical excision with other adjuvant treatment options of this rare pediatric disease would help in a better outcome.

## Highlights

• Langerhans cell histiocytosis can present as a mass in the temporal or parietal region with no symptoms otherwise.
• The etiology of Langerhans cell histiocytosis is still unknown and diagnosis can be challenging.
• The prognosis depends greatly on early identification and prompt surgical treatment.

## 1 Introduction

Langerhans cell histiocytosis (LCH) is a CD1a and/or S100 antigen-positive inflammatory disease consisting of characteristic Langerhans cells. The etiology of this proliferative disorder is unknown. LCH mostly presents in young adults and children, with male predominance [1], [2]. Pediatric LCH affects the head and neck region in 50-80% of cases while the involvement of temporal bone is seen only in 15 to 60% of cases [3]. In immunohistochemistry, characteristic features are the presence of inflammatory infiltrates of lymphocytes and large numbers of eosinophils, plasma cells and giant cells along with Langerhans cells. The gold standard method to diagnose LCH is through a histopathological study [4]. LCH has uncertain and complicated clinical features, thus resulting in misdiagnoses as inflammatory ear lesions and malignant tumors, ultimately with varied treatment and prognosis. Bone damage is the most common clinical presentation [5]. After diagnosis, depending on the severity of the disease, treatment options include surgery, chemotherapy, systemic steroids or combination therapy [6]. This work has been reported in line with the SCARE criteria [7].

Thus, this study aims to discuss and describe the clinical manifestations of LCH, its diagnostic modalities and the appropriate management.

## 2 Case report

A 10-year-old boy presented to our Neurosurgical outpatient clinic with a chief complaint of swelling on the right temporoparietal region, noticed by his parents after he sustained a fall from his bicycle. Since then, the swelling had been gradually increasing in size and was associated with pain in the local area. However, other clinical symptoms, such as fever, nausea, vomiting, headache, abnormal involuntary body movements, and loss of consciousness, were not present.

On presentation, vital signs were maintained, GCS was 15/15, and routine nervous system examination was normal. A single, 3 × 3 cm, non-tender, cystic, immobile swelling in the right temporoparietal region was noticed during the local examination. Subsequent ultrasonography of the scalp and the neck revealed a space-occupying lesion and an enlarged right cervical lymph node, respectively.

**Fig. 1:**
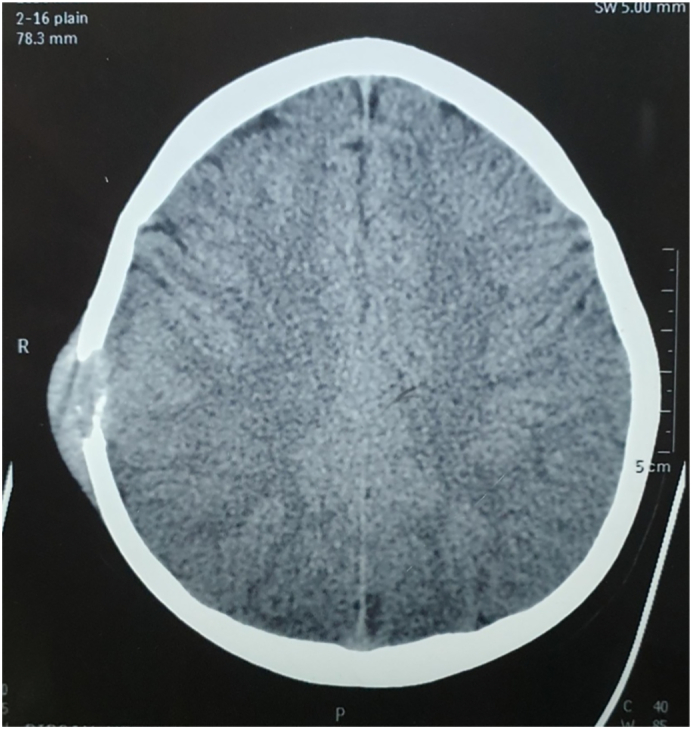
CT scan of the head revealed an approximately 3 cm sized defect involving both inner and outer table in the right temporoparietal calvarial with scalloped margin.

A computed tomography (CT) scan of the head revealed an approximately 3 cm-sized bony defect involving both inner and outer table in the right temporoparietal calvarium with scalloped margins as shown in Fig. 1. The defect consisted of well-defined soft tissue measuring 35x34x15mm with a density of +46 HU and bony fragments. There was no significant enhancement of soft tissue density in post-contrast images and no adjacent encephalomalacia changes. The CT scan was suggestive of eosinophilic granuloma found incidentally while the child was being evaluated for recent head trauma.

**Fig. 2:**
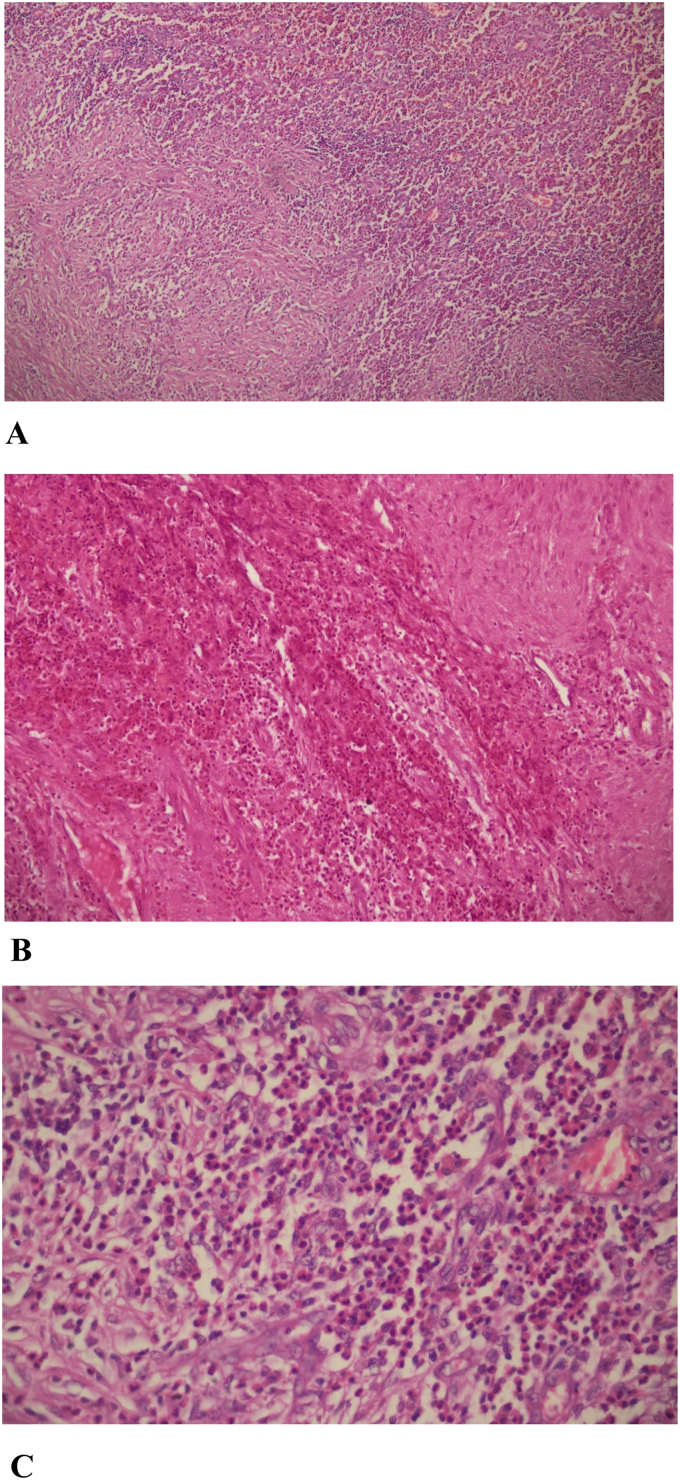
(A) Histopathological examination (100×). (B) Histopathological examination (200×). (C) Histopathological examination (400×). Showing singly scattered and a few clusters of histiocytes having round to convoluted nuclei while few histiocytes show nuclear grooving in the background of sheets of eosinophils, multinucleated giant cells, and foamy macrophages.

Fine Needle Aspiration Cytology was performed from the swelling over the right temporal area, and the aspirate was blood mixed. On microscopic examination, the smear showed predominantly singly scattered and a few clusters of histiocytes having round to convoluted nuclei. Few histiocytes showed nuclear grooving. The background consisted of sheets of eosinophils, multinucleated giant cells, and foamy macrophages. The FNAC was suggestive of a histiocytic lesion pertaining to a diagnosis of Langerhans cell histiocytosis as shown in Fig. 2(A, B and C). Plain and contrast-enhanced volume scan of the chest done from the lung apices to the upper abdomen showed a routine scan.

Thus, a diagnosis of LCH involving temporoparietal bone was made, and the patient subsequently underwent wide excision of the mass and cranioplasty.

Under aseptic precautions linear incision was given over the swelling, soft tissue dissection was done, surrounding pericranium was excised, the mass was resected en-bloc, and 2 cm of the surrounding bone margin was resected. The bony defect measuring 6 × 6 cm was replaced using bone cement. On macroscopic evaluation, a well-localized mass with minimal attachments with the dura and moderate vascularity was found. Moreover, the surrounding bone was soft with lysis of both the outer and inner table of the skull. A one-month follow-up CT scan of the head following excision of the mass showed bone cement graft in situ in the craniectomy defect with no evidence of residual or recurrent disease.

## 3 Discussion

LCH is a rare disease with variable clinical presentations, mostly seen in the pediatric age group.

Based on the age and clinical presentation, LCH is categorized into three variants. These variants include (a) Eosinophilic granuloma, a chronic localized form with skeletal involvement (single or multiple), mostly seen in adults; (b) Letterer-Siwe disease, an acute disseminated form with multiple system involvement often occurring mainly in infants; (c) Hand-Schuller-Christian disease, a chronic disseminated form with skeletal and extra skeletal lesions. Hashimoto-Pritzker disease, the congenital self-resolving form has also been reported [8]. In the afore-mentioned case, a diagnosis of eosinophilic granuloma is suggestive as the patient exhibited skeletal lesion and soft tissue swelling with no other extra skeletal involvement [9].

LCH is a rare disease with an incidence of 1 in 560,000. It is more common in males than females, with a reported ratio ranging from 1.1:1 to 4:1 [10]. The present case reported a 10-year-old male child. LCH may be due to reactive or neoplastic processes. Spontaneous remission and cytokine storm support reactive nature. Neoplastic nature is supported by clonal proliferation and association with the BRAF gene [11].

LCH can affect reticuloendothelial cells anywhere in the body. As such, multiple organs may be involved, most frequently the bones, skin, liver, lung, spleen, hypophysis, lymph nodes and bone marrow [12]. Solitary or multiple bone lesions are the most common clinical presentations seen in the majority of cases, with the most frequent sites involving the skull, ribs, vertebrae and pelvis. Bones of the skull commonly involved are orbital and temporal bones, sella turcica and mandible [13]. In the present case, the temporoparietal region of the skull was involved.

The most common symptom of eosinophilic granuloma is a gradually enlarging tender mass similar to the one present in our case [14]. Based on the clinical staging system proposed by Greenberg et al. in 1981, the present case is considered as stage 1a, as it involved a single bone with no extra skeletal manifestations [15].

Computed tomography (CT) scan is the preferred imaging modality for describing the extent and has a vital role in tracking disease activity and response to treatment [16].

The CT scan image generally has single or multiple, round to oval osteolytic lesions with sharp borders giving a punched out appearance of about 1–4 cm in diameter with indistinct bone margins in association with a homogeneous soft tissue mass. A “Double-contour” or “beveled edge” appearance due to involvement of both the inner and outer tables may be seen. The asymmetric beveling can be palpated beneath the scalp [17]. In this case, similar imaging findings were present.

Clinical diagnosis can be challenging as clinical and imaging findings are inconclusive, and tissue sampling via fine-needle aspiration or biopsy for histopathological examination may be required. LCH is diagnosed based on microscopic examination of the obtained biopsy specimens from the bony lesions. On microscopy, there is diffuse infiltration of sizable pale staining mononuclear cells with indistinct cytoplasmic borders and rounded or indented vesicular nuclei resembling histiocytes. Surrounding histiocytes are a varying number of eosinophils, lymphocyte, plasma cells. On electron microscopy, Birbeck granules, rod-shaped cytoplasmic structures within Langerhans cells are seen. On immunohistochemistry, the tissue stains positively for S-100 protein and CD1a [18].

Several treatment modalities for temporal bone LCH, including surgery, radiotherapy, chemotherapy, and steroid injection are used alone, or in combination, depending on the extent and severity of the disease [19]. Asymptomatic patients are kept under observation as some lesions resolve spontaneously [20].

In our patient, only a wide excision was performed because of unifocal involvement. Chemotherapy and/or radiotherapy should be considered if there was multifocal involvement. The prognosis of eosinophilic granuloma variant of LCH is excellent. Recurrence rates of around 1.6% to 25% are noticed, and a close and regular follow-up for an extended period is advised [10]. The 10 years survival rate for a single bone involvement is reported as 100%, while in a multisystem involvement, it is reported to be 77% [21].

## 4 Conclusions

This report highlights an incidental case of LCH in the right temporoparietal region in the pediatric age group as an unusual and commonly misdiagnosed case with no characteristic clinical features. Imaging and histopathology were found to be helpful in the diagnosis. Therefore, a careful assessment must be made with consideration to the LCH, when examining a swelling in the temporoparietal region with no other characteristic symptoms. The timely diagnosis and surgical excision with other adjuvant treatment options of this rare pediatric disease would help in a better outcome.

## Declaration of competing interest

None of the authors has any conflict of interest to disclose. We confirm that we have read the Journal's position on issues involved in ethical publication and affirm that this report is consistent with those guidelines.
